# Potential functions and applications of diverse microbial exopolysaccharides in marine environments

**DOI:** 10.1186/s43141-022-00432-2

**Published:** 2022-11-01

**Authors:** Hassan A. H. Ibrahim, Hala E. Abou Elhassayeb, Waleed M. M. El-Sayed

**Affiliations:** Marine Microbiology Department, National Institute of Oceanography and Fisheries (NIOF), Cairo, 11516 Egypt

**Keywords:** Microbial exopolysaccharides, Marine environment, Antifouling agents, Bioremediation

## Abstract

Exopolysaccharides (EPSs) from microorganisms are essential harmless natural biopolymers used in applications including medications, nutraceuticals and functional foods, cosmetics, and insecticides. Several microbes can synthesize and excrete EPSs with chemical properties and structures that make them suitable for several important applications. Microbes secrete EPSs outside their cell walls, as slime or as a “jelly” into the extracellular medium. These EPS-producing microbes are ubiquitous and can be isolated from aquatic and terrestrial environments, such as freshwater, marine water, wastewater, and soils. They have also been isolated from extreme niches like hot springs, cold waters, halophilic environments, and salt marshes. Recently, microbial EPSs have attracted interest for their applications such as environmental bio-flocculants because they are degradable and nontoxic. However, further efforts are required for the cost-effective and industrial-scale commercial production of microbial EPSs. This review focuses on the exopolysaccharides obtained from several extremophilic microorganisms, their synthesis, and manufacturing optimization for better cost and productivity. We also explored their role and applications in interactions between several organisms.

## Background

The marine biosphere is a heterogeneous mix of several ecosystems such as microbial mats, Antarctic Sea ice, hyper-saline marine environments, and shallow and deep-sea hydrothermal vents. Within the deep-sea hydrothermal vents, large physicochemical gradients exist. For example, the temperature of the surrounding seawater varies from 2°C to that of the hydrothermal plume, which can reach 350°C. Due to their microbial diversity, these ecosystems provide a wealth of novel biomolecules as several new microorganisms with highly diverse metabolisms have been isolated from these environments [1]. They offer vast natural resources for essential and functional commercial grade products such as EPSs [2].

Among the marine microbes, bacteria, and phytoplanktons, such as diatoms, cyanobacteria, and dinoflagellates, are the most significant sources of EPSs. Numerous EPS-producing microbes have been isolated from marine environments, such as seawater, sediment, deep-sea hydrothermal vents, and sea ice [3]. Marine microorganisms such as *Acinetobacter*, *Arthrobacter*, *Pseudomonas*, *Halomonas*, *Myroides*, *Corynebacteria*, *Bacillus*, and *Alteromonas* sp. have been studied for EPS production [2].

Commonly, EPSs are defined as natural weight polymers that are synthesized and secreted by microorganisms into their surroundings to establish the functional and structural integrity of biofilms. Hence, they are essential for determining the physicochemical properties of biofilms [4] and constitute 50–90% of a biofilm’s total organic matter [5]. Moreover, EPSs are mainly composed of polysaccharides and proteins, DNA, lipids, and humic substances [6, 7].

Microbial polysaccharides are principally classified into several groups based on (i) their cellular location (cell wall PSs, exoPSs, and endoPSs), (ii) structure (linear and branched), (iii) sugar composition (homo- and heteropolysaccharides), and (iv) type of linkages between monomers {b-(1→3), b-(1→6), and α-(1→3)} [8]. Based on their monomeric composition, the microbial EPSs are either homopolysaccharides, consisting of a single monomer linked by glycosidic bonds, or heteropolysaccharides, which have more than two monomeric units connected by glycosidic bonds. They also contain several different organic moieties, such as organic and amino acids, along with inorganic constituents such as sulfates and phosphates [9]. The polymers that belong to the homopolysaccharides group include cellulose, curdlan, dextran, pullulan, and scleroglucan [10]. Microbial EPSs can be further grouped into four major classes; polysaccharides, slime, and microcapsular polysaccharides, inorganic polyanhydrides (polyphosphates), polyamides, and polyesters [11].

Furthermore, EPSs are ideal for several applications due to their recently discovered chemical properties and structures [12]. Latest studies have shown antioxidant, immune-modulation, anti-tumor, and antimicrobial properties of EPSs [13].

Microbial EPS is an important source of dissolved organic carbon in marine ecosystems. Bacterial EPS are rich in uronic acid, which makes them resistant to mineralization by microbes and thus, can exist for a long time in oceans. Therefore, they are prevalent in extreme marine environments and are essential for microbial survival [14]. While EPSs mainly have protective functions, their exact roles depend on the microorganisms’ surrounding environment. They can protect the microbial communities against extreme temperature and salinity and lack of nutrient accessibility by forming a barrier between the microbe and its environment [13].

EPSs have different functions in bacteria, such as forming a favorable microenvironment to facilitate attachment, exoenzyme activity, sequestration of nutrients, and protection against toxins in the surrounding medium [3]. Additionally, they are essential for aggregate formation, surface adhesion, forming biofilms and biofouling, absorption of nutrients, and so on [15].

Due to their degradability and nontoxicity, microbial EPSs have attracted interest for their applications in the marine environment, especially as bio-flocculants [16]. Furthermore, they can be used as antifouling agents in wastewater treatment, bioremediation, and petroleum industries [17]. Therefore, this review aims to present comprehensive information on marine microbial EPSs, their sources, and potential prospective applications.

## Microbiology of EPS-producing marine organisms

EPSs have been primarily observed in terrestrial and marine bacteria and fungi [17], and occasionally, in yeasts [18], cyanobacteria [19], microalgae [20], and medicinal mushrooms [21]. The following sections will briefly summarize the common microbes that produce EPSs, starting with extremophilic microorganisms.

### EPSs from archaea and bacteria

Different types of EPS have been isolated from different groups of archaea, especially thermophilic and halophilic groups. Thermophilic archaea have been isolated from extreme environments, including deep and shallow marine hot springs and terrestrial hot springs [22]. Various thermoacidophilic archaea, including members of the genera *Thermococcus* and *Sulfolobus*, have been reported to store polysaccharides, such as glycogen, and secrete mannan and sulfated heteropolysaccharides [23]. Significant accumulation of EPSs was observed in *Archaeoglobus fulgidus* and *Archaeoglobus profundus* in the form of biofilms [24]. Different strains of thermoacidophilic archaeon, *Sulfolobus solfataricus*, were used to produce sulfated EPSs [25]. Moreover, two closely related hyperthermophilic crenarchaea, *Sulfolobus acidocaldarius* and *S. tokodaii*, were studied by Koerdt et al. [26] for biofilm formation.

**Fig. 1:**
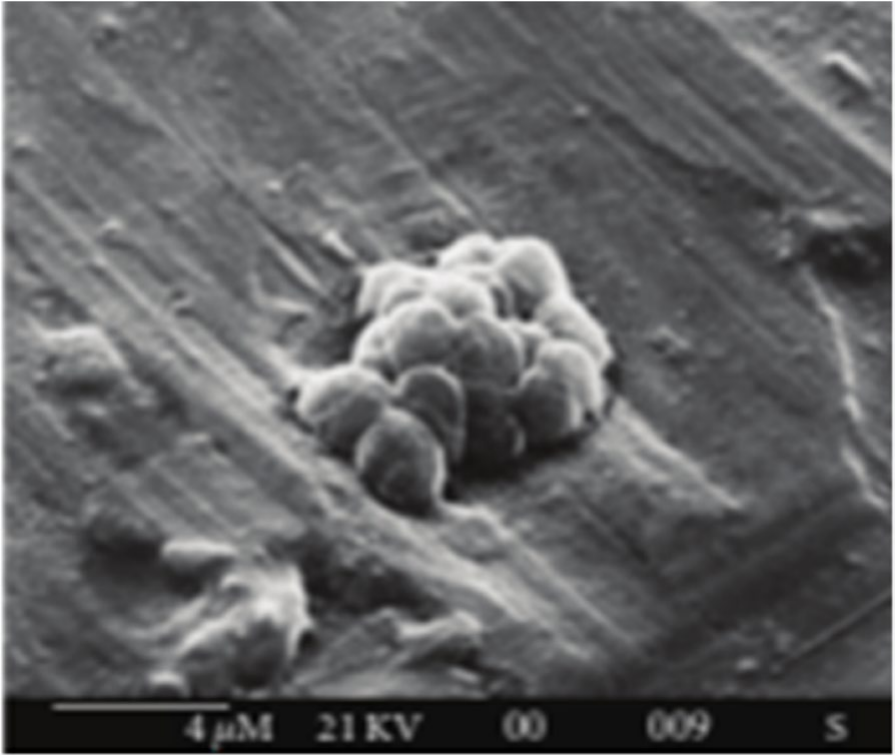
Biofilm production by *H. japonica* T5 [31]

The EPSs synthesized by *Halomonas* strains had high sulfate content and a considerable amount of uronic acids showing high gelation capability [27]. However, Anton et al. [28] produced a heteropolysaccharide EPS using an archaebacterium, *Haloferax mediterranei*. Paramonov et al. [29] elucidated the neutral structure of EPS isolated from *Haloferax gibbonsii*. Furthermore, Parolis et al. [30] separated an acidic EPS from a halophilic archaeon, *H. denitrificans*. Moreover, Nicolaus et al. [31] isolated an obligate halophilic archaeon, *Haloarcula japonica* T5, that produces a sulfated EPS (Fig. 1). according to the previous literature, many microorganisms produce exopolysaccharides as a strategy for growing, adhering to solid surfaces, and surviving adverse conditions. The physiological role of EPS depends on the ecological niches and the natural environment in which microorganisms have been isolated.

**Fig. 2:**
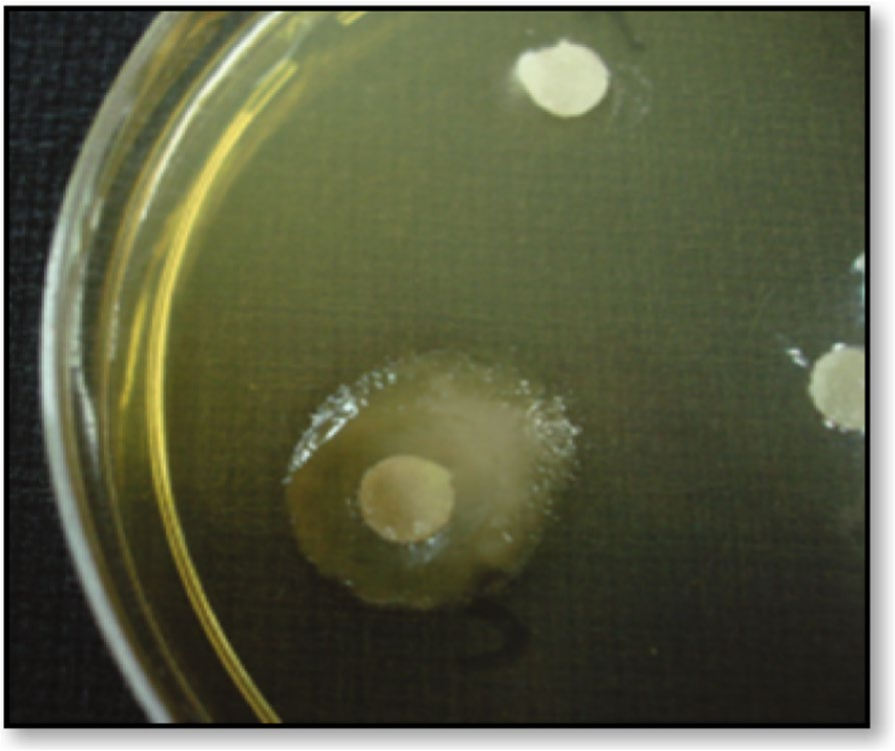
Mucoid colony of an exopolysaccharide-producing microbe on solid media [34]

Interestingly, most bacterial species grown under appropriate culture conditions secrete mucoid polysaccharides outside the rigid cell wall structures [32]. However, the presence of EPS in bacterial cells can easily be identified by the appearance of the mucoid colony, as shown in Fig. 2 [33].

Studies on EPS focus mainly on the polysaccharides produced by Gram-negative and some Gram-positive bacteria [35], such as *Pseudomonas* spp., *Acetobacter* spp., *Aureobasidium* spp., *Sinorhizobium* spp., *Escherichia* spp., *Acetobacter* spp., *Bacillus* spp., etc. [36]. Enos-Berlage and McCarter [37] showed that *Vibrio parahaemolyticus* secrete EPS.

Ravaioli et al. [38] screened 55 *S. epidermidis* biofilm-forming clinical isolates using a simple fluorescence-based microtiter-plate assay. Several species from the genus *Enterobacter* secrete EPS-containing fucose, such as *Enterobacter* sp. CNCM 12744, that produces EPS-containing fucose, galactose, glucose, and glucuronic acid monomers [39]. Freitas et al. [40] found that *Enterobacter* strain A47 (DSM 23139) produced a fucose-containing EPS. Lactic acid bacteria (LAB) are mesophiles that have been long known to produce EPS. Among their genera, *Lactobacillus bulgaricus, L. helveticus*, *L. brevi*, *L. lactis*, *Leuconostoc mesenteroides*, and *Streptococcus* spp. are the potent EPS producers. Also, more than 30 LAB species are polysaccharide producers, of which *Leuconostoc mesenteroides* is a commercially used dextran producer [41].

Marine bacteria have been reported to produce a wide range of EPSs. Isolating new EPS-producing bacteria from marine environments, particularly extreme ones, has been of interest [42]. Jayaraman & Seetharaman [43] isolated EPS from the marine bacterium, *Vibrio alginolytics*, which acted as a potential marine biofouling material. Similarly, the marine bacteria, *Vibrio diabolicus* produces hyaluronic acid-like EPS that has been commercialized with the trade name, “Hyalurift.” Amazingly, this EPS can improve bone integrity [44]. Gutierrez et al. [45] isolated a type of EPS, also known as PE12, with emulsifying activity from *Pseudoalteromonas* that can adsorb metal ions. Urai et al. [46] isolated marine *Rhodococcus erythropolis*, PR4 that produces many acidic EPSs including FR2. Additionally, the EPS-producing *Pseudoalteromonas* sp. strains, CAM025 and CAM036 were isolated from seawater and sea ice in the Southern Ocean [47]. CAM025 showed 30 times higher yield when grown at −2°C and 10°C than at 20°C. Al-Nahas et al. [48] isolated a marine EPS-producing *Pseudoalteromonas* sp from the Red Sea sponge found in Egypt.

Selim et al. [49] recently examined 83 marine isolates (from the Mediterranean Sea and the Red Sea) for EPS production. Of these, nine isolates showed the highest antioxidant activities; *Bacillus circulans*, *B. licheniformis*, *B. alvei*, *B. insolitus*, *B. polymyxa*, *B. marinus*, *B. anthracis, Staphylococcus sp.,* and *B. brevis*. El Essawy et al. [50] extracted an EPS from marine *Klebsiella* sp. Abdelnasser et al. [51] isolated EPS-6 from the bacterial strain, *Bacillus flexus* from the Mediterranean Sea. Wang et al. [14] produced EPS-A from the marine bacteria, *Aerococcus uriaeequi*. The EPS extracted by Abdrabo et al. [52] from marine *Halomonas saccharevitans* AB2 isolated from the Suez Gulf, Egypt, showed promising antimicrobial and anti-tumor activities. Selim et al. [53] isolated 20 streptomycetal strains from marine sediment samples collected from the Nabq area, Red Sea, Egypt. EPS exhibiting potent anti-tumor activities were produced in vitro using four strains, particularly *Streptomyces carpaticus*. Ali et al. [54] optimized EPS production from marine *Pseudomonas mendocina AB1*, emphasizing valuable applications such as antioxidant and antibacterial agents*.*

Amazingly, thermophilic bacteria, derived mainly from hydrothermal vents and hot springs, including *Archaeoglobus fulgidu*, *Thermococcus litoralis*, *B. thermantarcticus*, *Geobacillus thermodenitrificans*, *B. licheniformis*, *Thermotoga maritima*, *Thermoto gamaritima*, *Methanococcus jannaschii*, and *Geobacillus tepidamans* V264 hare well-documented EPSs producers [36].

Marine bacteria produce many EPSs in numerous ways. The genes for EPS synthesis are frequently found in clusters inside the genomes of the EPS-producing organisms. Further development of genetic, metabolic, and protein-engineering techniques is required to understand the underlying mechanisms involved in EPS production. This will also enable tailor-making EPS-based polymers with improved qualities for medicinal and industrial use. Novel applications can be developed by exploiting the natural design space for biopolymer synthesis [17].

### EPSs from marine fungi and yeast

Ascomycota and Basidiomycota fungi can be used to produce several synthesized EPSs with unique biochemical and biological properties [55]. These EPSs are mainly heteropolysaccharides, but in the case of homopolysaccharides, glucose is their only monomer [22]. Also, there are several EPSs produced by filamentous fungi such as *Botryosphaeria rhodina* MMGR [56], *Aspergillus versicolor* LCJ-5-4 [57], *Fusarium solani* SD5 [58], *F. oxysporum* Y24-2 [59], and *Penicillium griseofulvum* [60]. Moreover, a coral-associated fungus, *Penicillium commune* produces EPS, FP2-1, when grown on potato dextrose agar medium [61].

Actually, EPSs are produced by several yeasts such as *Candida*, *Candida famata* and *Candida guilliermondii* [62]; *Cryptococcus*, *Cryptococcus flavus* and *Cryptococcus humicolus* [63, 64]; *Lipomyces*; *Pichia* [30]; *Rhodotorula*, *Rhodotorula acheniorum* MC [65]; *Issatchenkia orientalis* [63]; *Kazachstania unispora* [66]; and *Sporobolomyces* genera such as *Sporobolomyces salmonicolor* AL1 [67]. Additionally, Kuncheva et al. [65] produced mannan from the yeast strain, *R. acheniorum* MC, and glucomannan from *S. salmonicolor* AL_1_. Pavlova et al. [64] applied psychrophilic Antarctic yeast, *C. flavus*, to produce the heteropolysaccharide EPS, composed of mannose, glucose, xylose, and galactose. Rusinova-Videva et al. [68] also isolated an EPS-producing psychrophilic yeast isolate.

### EPSs from marine cyanobacteria and algae

**Fig. 3:**
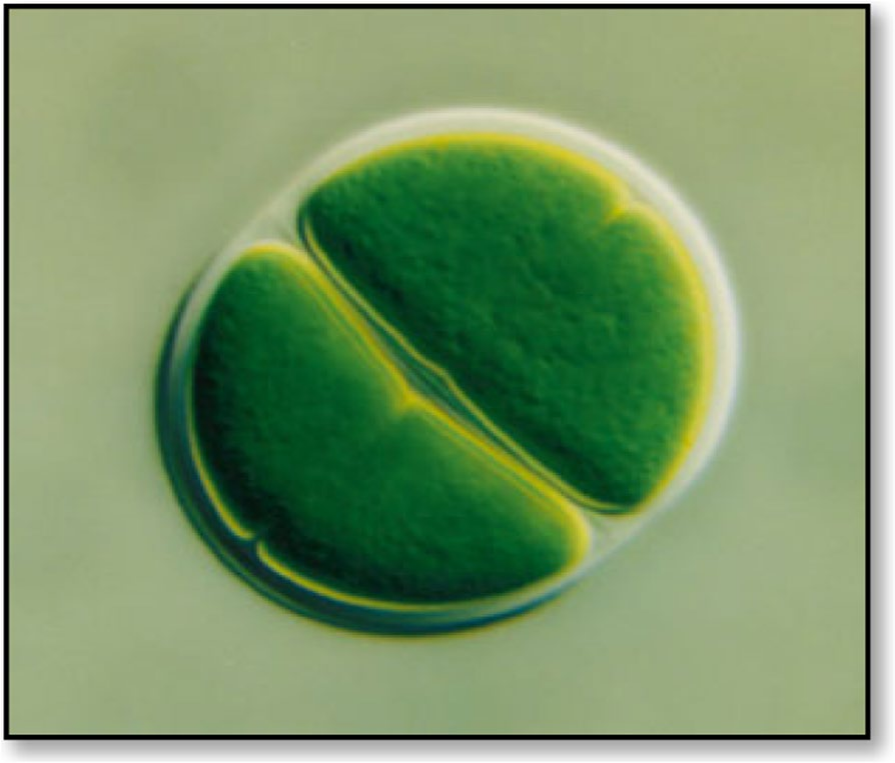
The structure of a “sheath” of the unicellular *Chroococcus* sp. [71]

**Fig. 4:**
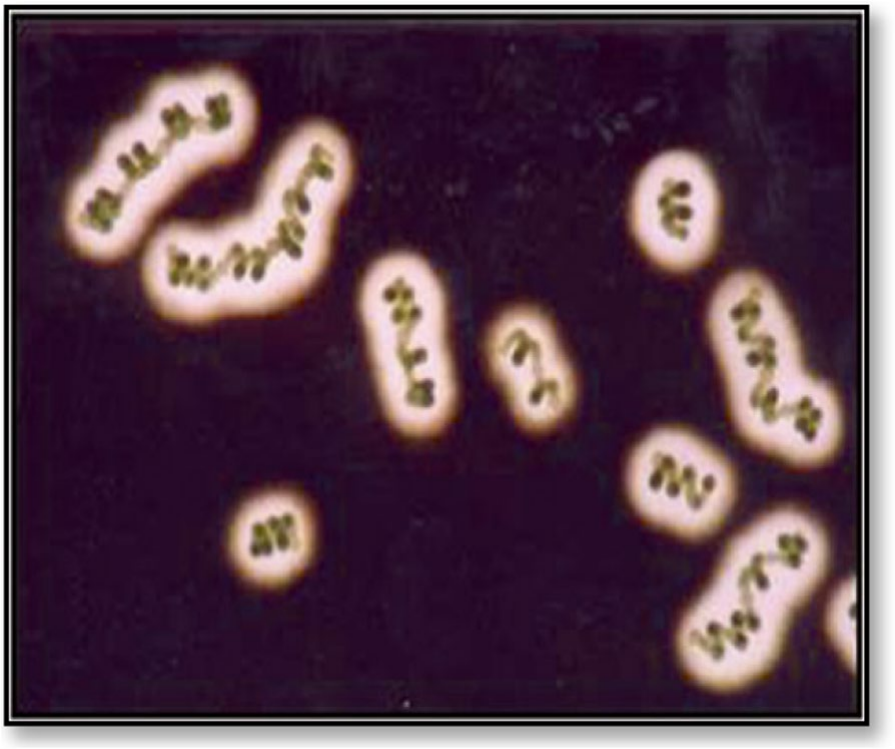
The exopolysaccharides of *Cyanospira capsulata* [22]

Cyanobacteria and green algae are phototrophic microorganisms with diverse cellular characteristics that change in response to the environmental conditions, such as producing EPS in response to harsh conditions. EPS is primarily found in the enclosed layer surrounding their cells/filaments and is then released into the environment. Generally, EPSs are vital for their survival under stress conditions like radiation, desiccation, and high temperatures. Microalgae and cyanobacteria EPSs are visible as a mucosal mass surrounding the cells [69]. They can closely adhere to the cells and be released into the surrounding medium [70]. They can be seen in a thin layer, known as a sheath (Fig. 3), which is formed adjacent to the outer cell membrane in the form of a capsule [22]. It is associated with the cell surface and may be covalently bound to the cell wall. When they are loosely associated with the cell surface and not within envelopes, they are considered as slime (Fig. 4).

De Philippis and Vincenzini [71] characterized several EPS-producing *Cyanothece* strains isolated from saline environments. Additional acetyl, pyruvyl, and sulfate groups have also been detected in some EPS samples. They facilitate cell adhesion by assembling as a stalk aside from providing structural support as capsules or sheaths [72].

In cyanobacteria, EPS and slime represent most of the cell’s dry weight, while sheaths represent a relatively smaller portion [73]. Although many cyanobacteria have been shown to produce EPSs, most of them were isolated from the terrestrial environment. Only a few marine cyanobacterial strains, such as *Schizothrix* sp., *Gscillatoria* sp., *Cyanothece* sp. [74], and *Oscillatoria* sp., isolated from marine stromatolites, have been documented for EPS production [75]. Additionally, studies suggest that *Spirulina* sp. produces several compounds containing polysaccharides and EPSs with therapeutic functions such as anti-inflammatory properties [76]. For example, spirulan is a sulfated EPS produced by *Arthrospira platensis* [44].

In red microalgae, the EPS is partly dissolved in the growth medium and partly released into the medium, increasing its viscosity. The soluble EPSs produced by red microalgae are either released from the bulk fraction or are transferred directly from the cell to the growth media [77].

## Production and characterization of EPSs

Microbes are more potent and cheaper sources of EPSs than plants because of their high growth rate, ability to grow in relatively affordable media, lower space requirement, and ease of manipulation. Thus, there is increasing interest in isolating and identifying novel microbial EPS that can compete with traditional EPS [78]. The research on EPSs primarily focuses on their synthesis, optimization of production to make it cost-effective, and finally, understanding their role and application in interactions between numerous organisms. Using biotechnological techniques, it is possible to obtain substantial amounts of EPSs from numerous microbes by controlling their growth conditions in a bioreactor [79].

**Fig. 5:**
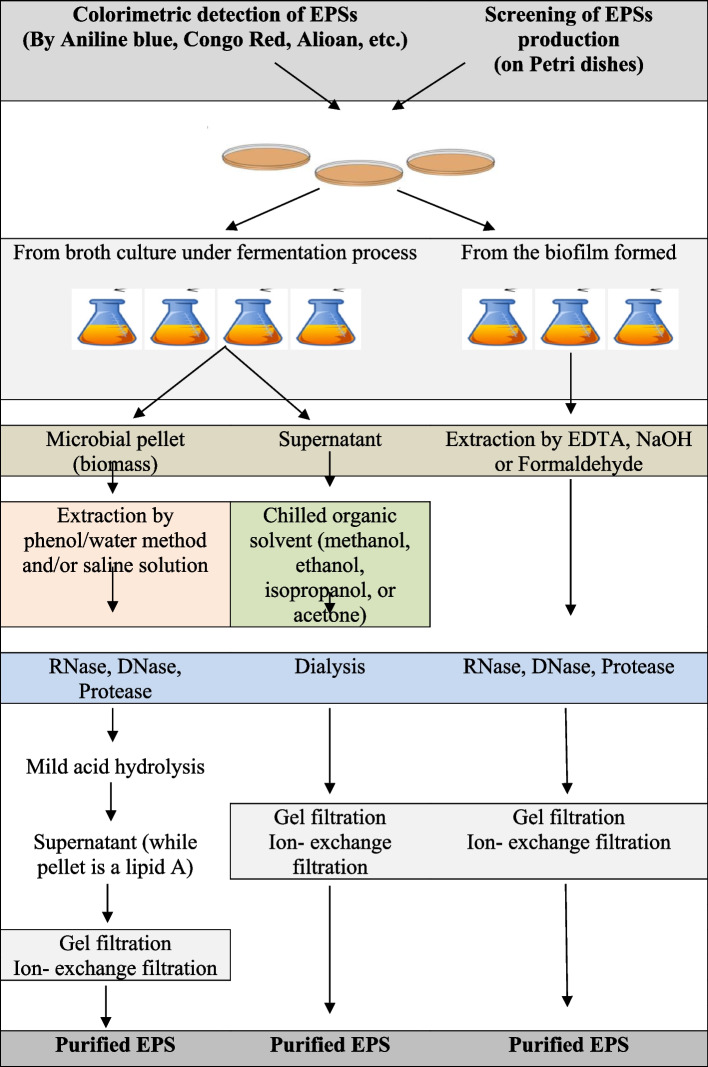
The general steps for production, extraction, and characterization of exopolysaccharides

First, a specific bacterial strain is examined for its possible EPS-producing ability to evaluate EPS production by observing if it is sticky or ropy. When liquid cultures of EPS-producing bacteria demonstrate high resistance to flow through serological pipettes and form viscous strands during free-fall from the pipette tip, they are considered “ropy” [80]. EPS production can be improved by developing novel strategies such as fermentation using genetically engineered microbes and methodologies resulting in high yield and cost-effective production. The downstream process to recover the EPS is conventionally done by removing the cells from the fermentation broth by centrifugation, followed by isolation and purification steps. Subsequently, the EPS present in the cell-free medium is precipitated using ethanol, methanol, or acetone. Then, the pellets that are recovered using centrifugation, dialyzed using the appropriate method in distilled water, are freeze-dried to obtain crude EPS [81]. The general steps of production, extraction, and characterization of EPSs are illustrated in Fig. 5.

Overall, EPS production involves selecting suitable microbes, the cultural media, and the practical method for EPS preparation and extraction. The isolation method employed can also significantly affect the EPS yield. Several physical and chemical methods have been applied to extract EPS from different sources, such as cell suspensions, sludge, biofilm, solid surfaces, and various types of water. The physical methods include centrifugation, sonication, heating, and freeze-thawing, while the chemical methods involve different chemical agents, such as organic solvents, NaOH, ethylenediamine tetraacetic acid (EDTA), and formaldehyde [61].

To isolate crude EPS, the supernatant is usually precipitated using alcohols such as ethanol (95%) or methanol and, occasionally, isopropanol or acetone at 4 °C for 12–24 h. Similarly, fungal EPSs are derived via ethanol precipitation using different ratios of the culture/water suspension and alcohol [82]*.* Occasionally, in strains like *Ascomycota* strains, the supernatant containing the EPS is treated with 5% trichloroacetic acid [83] during primary purification. The crude EPS is dialyzed against water to remove excess salts and then stored as a vacuum-dried or lyophilized powder. The next step involves using Sevage reagent to deproteinize the EPS for further purification [57]. Moreover, there are other potent methods to purify EPS from *Ascomycota* and *Basidiomycota* EPSs, such as ion exchange chromatography and gel permeation chromatography [82]. Cyanobacterial EPSs can be more easily recovered by simply precipitating the cell-free supernatants with cold ethanol [84]. Other methods include sheath extraction from *Chroococcus minutus* SAGB.41.79 with differential sucrose gradient centrifugation using homogenized cells. Some studies used hot water treatment of the pelleted cells, while others performed deionized water extractions to extract EPS. In other cases, cyanobacterial EPSs were isolated by treating pelleted cells with 1.5% NaCl at 60°C [85].

Furthermore, Freire-Nordi et al. [86] extracted EPSs from *Staurastrum inversenii* by fixing medium-starved cells with 0.5% formalin followed by progressive 4% Dakin liquid washes followed by stirring for 30 min at 40°C. Di Pippo et al. [84] recovered cyanobacterial EPSs by extracting with 0.1 M H_2_SO_4_ at 95°C for 1 h. Abdullahi et al. [87] used part water at 90°C with 0.5 M NaHCO_3_ at 95°C and part 1 M NaOH containing 0.2 M NaBH_4_ at 95°C to extract the bulk mucilage from the fungal diatom, *Phaeodactylum tricornutum*.

For characterizing microbial EPS, the basic parameters that should be analyzed include the total content of carbohydrates, uronic acids, sulfated sugars, and protein that can be determined by standard methods [88]. Additionally, EPS hydrolysis using acids or other agents, including sulfuric acid, hydrochloric acid, trichloroacetic acid, and trifluoroacetic acid, should be done to break down the glycosidic linkages of the polymer and subsequently expose the monosaccharide constituents. These monomers are reduced to form sugar alditols and further derivatized by acetylation with acetic anhydride in the presence of pyridine. These volatile sugar derivatives are then subjected to gas chromatography-mass spectrometry (GC-MS) analysis and compared with the sugar standards [22]. Furthermore, several advanced methods have been approved for the qualitative analysis of EPSs, including high-performance liquid chromatography, Fourier transform infrared spectroscopy (FTIR), and nuclear magnetic resonance [89]. Recently, matrix-assisted laser desorption/ionization time-of-flight mass spectrometer (TOF), atomic force microscopy, and X-ray diffraction have also been used for the detailed qualitative analysis of EPSs [90].

## Ecological roles of microbial EPS in the marine environment

EPS constitutes most of the ocean’s reduced carbon storage and supports the survival of marine bacteria by changing the physicochemical environment around the bacterial cell. Furthermore, they protect microbial communities against extreme temperature, salinity, and nutrient availability extremes [10]. The physiological role of EPS is dependent on the ecological niches and the natural environment in which microbes have been grown. EPS are mainly essential for aggregate formation and adhesion to surfaces. In certain organisms, they are required for biofilm formation and biofouling, along with the absorption of nutrients [15]. The critical roles of EPSs are discussed in the following sections.

### Aggregation of microbes

In their natural environment, most bacteria occur in microbial aggregates, and their structural and functional integrity depends on the presence of a matrix made of extracellular polymeric substances including EPS. Hence, EPS production is essential for their survival. Particularly, the organic matrix present in the intracellular space of microbial biofilms, which represents a significant store of reduced carbon on Earth, is made up of marine polysaccharides and other macromolecules such as proteins, lipids, and nucleic acids. Furthermore, the latest focus on extreme marine habitats has increased awareness for the bacteria surviving in these environments, known as extremophiles. These species serve as a model for studying the stability and potential function of their biomolecules due to their unique metabolic pathways and defense systems [10].

Undoubtedly, they have protective functions by forming a layer around the cell and providing adequate protection against high or low temperatures, salinity, and possible predators. They are essential for aggregate formation, adhesion to surfaces and other organisms, the formation of biofilm, and uptake of nutrients [91]. Notably, studies involving the microbial communities in sea ice have found strong associations between bacteria and particles, indicating the importance of EPSs in cryoprotection [92].

### Formation of bacterial biofilm

The ability to build and maintain an organized multicellular bacterial population highly depends on the production of extracellular matrix components [93]. Although the biofilm matrix might consist of several molecules, we have focused on EPSs critical for biofilm development in this section. The biofilm-forming bacteria are more protected than the planktonic bacteria as the EPS matrix acts as a protective diffusion barrier [94]. Furthermore, due to its gluey nature, the EPS layer serves as a nutrient trap, facilitating bacterial growth [95]. Thus, these polymers are the primary components of the biofilms formed on solid substrates [96]. Furthermore, reports have shown that the biofilm-forming microbes are more than 1000 times resistant to antibacterial compounds such as antibiotics, toxins, surface active agents, and sanitizers than free planktonic cells. Therefore, EPS formation is crucial for the survival of these microbes [97].

A bacterial biofilm is “a structured community of bacteria encapsulated within a self-developed polymeric matrix and adherent to a living or inert surface.” It is often characterized by surface attachment, structural heterogeneity, genetic diversity, complex community interactions, and an extracellular matrix of polymeric substances [98]. In nature, bacteria colonize at various interfaces to form poly-bacterial aggregates such as mats, flocs, sludge, or biofilms, unlike planktons that are dispersed, single cells as seen in pure laboratory cultures [99].

**Fig. 6:**
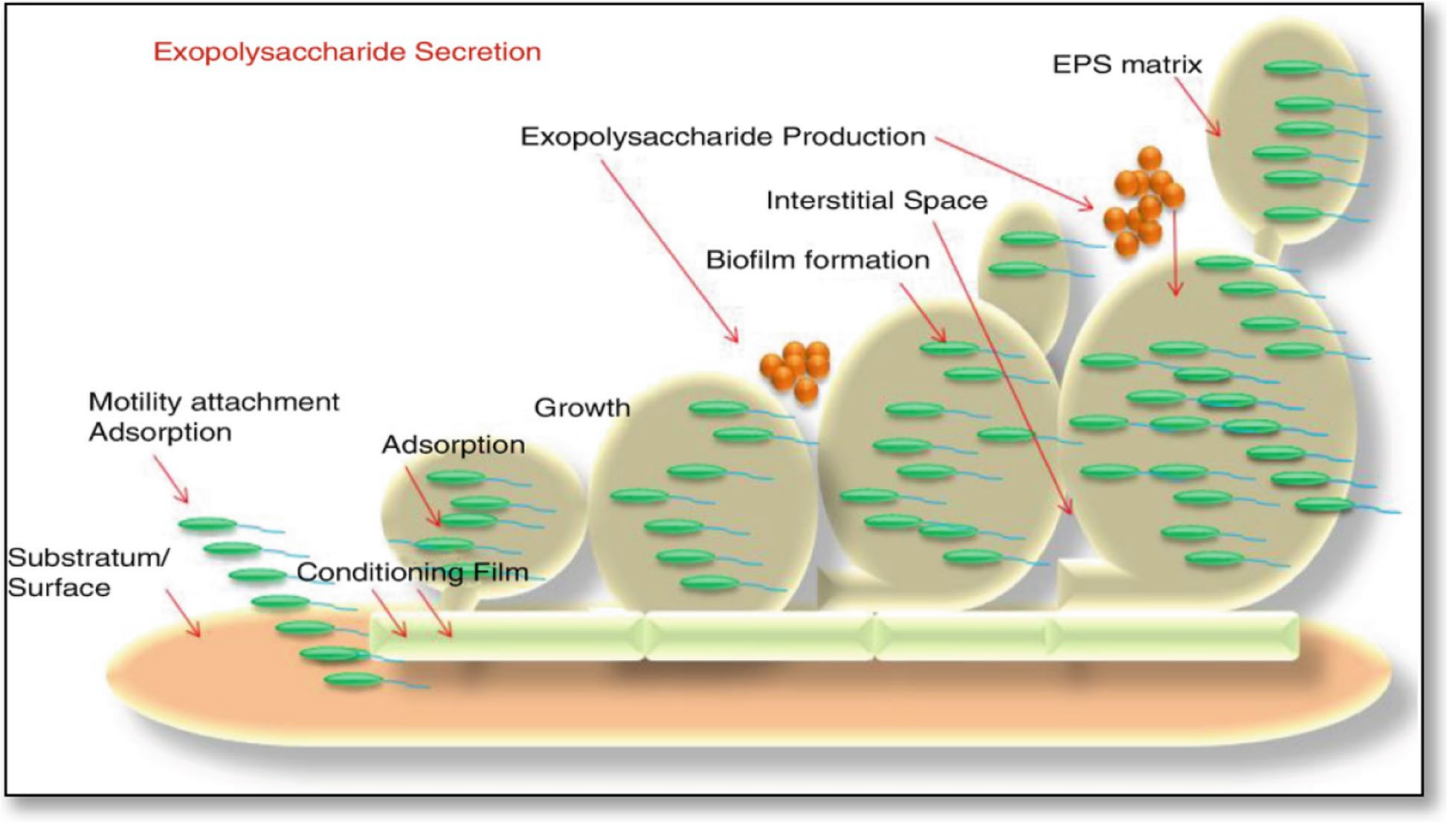
General steps involved during the formation of the exopolysaccharide matrix of a biofilm

Several essential elements influence the process of bacterial biofilm formation. Water quality, including temperature, pH, dissolved oxygen level, and the presence of organic and inorganic nutrients, is highly significant. After finding a suitable environment, the bacterium will continue to develop unless the system’s conditions become unsuitable [100]. EPSs is essential for the biofilm matrix-mediated biochemical interactions between the bacteria and its surrounding cells. Hydrated biofilms offer a stable microenvironment for storing extracellular enzymes and for the cellular uptake of small molecules [101] (Fig. 6).

Unlike the adhesion seen in biofilms, EPS are integral to interactions such as cell-cell cohesion and cell-solid substratum cohesion [102], which allow the bacteria to colonize densely in their hosts and microenvironment along with protecting them from harm. Therefore, understanding biofilm dynamics is crucial for developing novel and effective biofilm suppression control measures to improve desalination management [103].

The rate of synthesis and the number of EPS accumulated in the capsules in pathogenic bacteria influence their pathogenicity. Besides, EPSs plays an important role in the biofilm matrix by enabling biochemical interactions between the bacteria and its surrounding cells [104]. Hydrated biofilms offer a stable microenvironment for storing extracellular enzymes and for the cellular uptake of small molecules [67].

However, there are several critical drawbacks of EPSs as follows:

1. They act as starters for biofilm growth inside water pipes, affecting water quality by changing the bacterial levels (increasing coliform bacteria), reducing dissolved oxygen, and changing the taste and odor.
2. It provides a platform for biofouling in aquatic systems, such as organisms, ship hulls, pipelines, and reservoirs [17]. However, a collective summary of the potential roles of EPSs in bacterial biofilms is presented in Table 1.

**Table 1:** A side of the roles accompanied to exopolysaccharides in the biofilms [105]

Phenomenon	Functions of EPSs related to biofilms
Adhesion	EPSs make provision for the initial steps in the colonization of surfaces by abiotic, biotic, and long-term attachment of the biofilms.
Bacterial cell aggregation	The bridging between cells is enabled by EPSs, leading to the subsequent development of high cell densities and cell-cell recognition.
Water retention	Hydrophilic EPSs have high water retention ability thus maintaining a hydrated microenvironment around the biofilm leading to the survival of desiccation in water-deficient environments.
Cohesion of biofilms	Neutral and charged EPSs form a hydrated polymer network, known as the biofilm matrix, mediating the mechanical stability of biofilms, determining the biofilm architecture, as well as allowing cell-cell communication.
Nutrient source	EPSs serve as source of carbon, nitrogen and phosphorus containing compounds for utilization by the biofilm community.
Protective barrier	EPSs confer resistance to non-specific and specific host defenses during infection. They confer tolerance to various anti-microbial agents and protect cyanobacterial nitrogenase from the harmful effects of oxygen and offers protection against some phagocytic protozoa.
Sorption of organic compounds and inorganic ions	Charged and hydrophobic EPSs mediate the accumulation of nutrients from the environment, sorption of xenobiotics and recalcitrant materials. They promote polysaccharide gel formation resulting in ion exchange, mineral formation and the accumulation of toxic metal ions, contributing collectively to environmental detoxification.
Sink for excess energy	EPSs stores excess carbon under unbalanced carbon to nitrogen ratios.
Biofouling formation	By accumulating the microorganisms that able to secret EPSs on wetted surfaces or vehicles, then followed by plants, algae, or animals.

### Architecture and featuring of the marine environment

EPS matrix molecules provide a three-dimensional framework that allows cells to localize extracellular activities and perform cooperative/antagonistic interactions that are unachievable in free-living cells [106]. In a geomicrobiological environment, EPSs influence precipitation of minerals, mainly carbonates. They might also be able to trap various particles in biofilm suspensions, limiting dispersion and element cycling [107]. Furthermore, EPSs improve sediment stability by affecting sediment cohesion, permeability, and erosion (Table 1). The adhesion and metal-binding ability of EPS affect mineral leaching rates in both environmental and industrial contexts. These interactions between EPSs and the abiotic environment allow them to primarily affect the biogeochemical cycling [107]. EPS alters the optical fingerprints of sediments and saltwater and is involved in biogeomineral precipitation, microbial macrostructure creation, and horizontal genetic information transfer [106].

### Formation of marine snow and biological bump

**Fig. 7:**
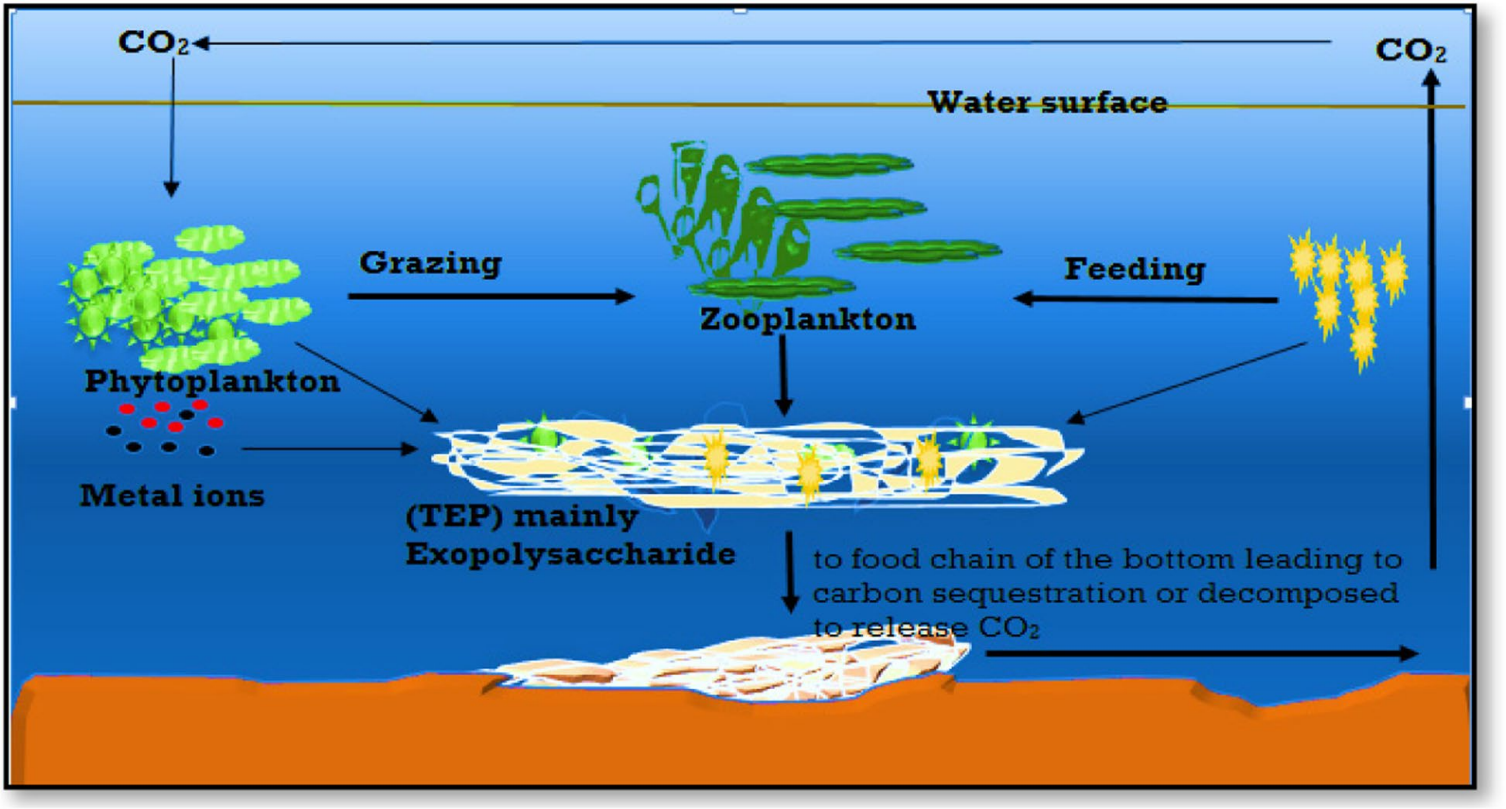
Aggregation of organic carbon by exopolysaccharides and scavenge some metal ions then sink to the bottom [108]

EPSs enable the production of organic colloids and large cell aggregates known as marine snow. These include transparent exopolymer particles, sea-surface microlayer biofilm, and marine oil snow [106]. Excessive EPS formation occurs as a metabolic byproduct during the late phases of phytoplankton blooms, releasing a carbon pool that alternates between dissolved, colloidal, and gel phases. However, the coagulation of single particles into rapidly settling aggregates is known as a “biological bump” [108] (Fig. 7).

In addition, Krembs et al. [109] demonstrated that prevailing under-ice currents and ice drifts carry the neutrally buoyant polymeric materials over long distances. Further evidence has indicated the importance of marine bacterial exopolymer synthesis in the process of aggregate formation [104]. When this colloidal material was released into the water column, a combination of biological, chemical, and physical pressures enables its congregation and formation of microbiological heterotrophic activity centers [110].

### Formation of biofouling

Biofouling is a natural phenomenon that occurs when biofilms form on water-immersed surfaces. Biofilm formation is the first and most crucial step in the biofouling process. Biofilms undergo several stages, including substratum conditioning, pioneer bacterial adherence, extracellular polymeric material release, soft macrofouling, and hard macrofouling. Biofouling has a financial impact on industries. Biosurfactants have antibacterial and biocompatible qualities, making them attractive antifouling solutions for biofouling processes. EPSs are integral for the biofouling formation in the aquatic systems and on the marine vehicles affecting vessels and other marine structures. However, biofouling starts with a layer of adsorbed organic and inorganic matter, through microbial film formation, to a community of macroscopic plants and animals (see Table 1) [17].

Studies showed that the addition of EPS-producing *Nostoc muscorum* into the soil increased the number of water-stable aggregates, either by gluing the soil particles or by stimulating the soil community to produce more EPSs [111]. The function of the hydrophobic EPSs in the adhesion process was also supported by the observation that treatment with the sulfated polysaccharide, emulcyan excreted from *Phormidium* sp. that masks the hydrophobicity of EPSs, caused cell detachment from solid surfaces. Benthic cyanobacteria *Phormidium* J-1 and *Anabaenopsis circularis* 6720, which could co-flocculate with suspended clay particles and attach to the benthos due to the hydrophobic interactions. In marine environments, cyanobacterial and diatom-produced EPSs form a matrix that enhance mudflat sediments by stabilizing them to against erosion and enriching them with organic matter and nutrients [112].

Biological fouling is a serious issue for constructed structures in marine and freshwater environments as microbial biofilm formation are frequently followed by colonization by several macro fouling organisms. Use of antimicrobial chemicals, usually poisonous to non-target species, is a common and effective preventative strategy. While various nontoxic surface modification approaches have been used, their effectiveness in in situ situations has been limited.

Marine microorganisms are important potential sources of antifouling chemicals due to their diverse metabolic activities and specific structural moieties. Chemical substances can disrupt the biofilm’s bacterial structure and interfere with higher organisms’ larval settling [113]. As only a small percentage of microorganisms have been found to contain bioactive substances, further research should be done to isolate and grow bacteria, especially those from harsh habitats. Aerobes, mesophiles, and heterotrophs have been found to be non-picky EPS producers isolated from hydrothermal vents [114].

Conversely, microbial EPSs used as biosurfactants and bioemulsifiers have attracted attention because of their biodegradability [115]. Therefore, a promising technology, known as microbial enhanced oil recovery (MEOR), has been developed for manipulating the function and structure of microbial environments in oil reservoirs. It is a biotechnology method in which microbes could be used to recover the additional oil from existing wells, thereby enhancing the petroleum production from an oil reservoir [115]. However, selected natural microbes producing biosurfactants and/or specific EPSs are introduced into oil wells to produce harmless by-products, such as slippery natural substances or gases, all of which aid the oil expulsion out of the well, allowing higher quantities to be recovered from the well. Genetically engineered *Enterobacter cloacae* are successfully used in MEOR [115].

### Tolerance to water stress and UV radiation

UV-B/A irradiation damages live cells by producing reactive oxygen species (ROS), which mostly include superoxide anion (Radical dot-O_2_) and the hydroxyl radical (Radical dot-OH) [116]. Hydroxyl and superoxide radicals are two major free radicals that can directly cause a variety of oxidations. Antioxidation may be disrupted if hydroxyl or superoxide radical scavengers are present, allowing free radical scavengers to protect living cells from UV radiation. Free radical scavenging properties have been discovered in a variety of polysaccharides [117, 118].

The protection against water stress and UV radiation is one of the main studied roles of the EPSs in constrained environments. It is known that the cyanobacteria isolated from very dry environments, such as desert soils or the lithic surfaces of monuments, display the capacity of excreting large amounts of EPSs [119, 120], a trait underlining adaptation to drought. Dehydration effects have been thoroughly studied. Essentially, water stress leads to the loss of membrane structural integrity and the loss of macromolecule functioning [121], so some authors associate cell death under drought conditions just with the loss of membrane integrity [122].

Although the role of EPSs in water stress has not been fully clarified, they are reportedly involved in maintaining hydration thanks to their hydrophilic/hydrophobic characteristics, which determine a gelatinous envelope around the cells that regulates water uptake and water loss processes [123]. Furthermore, they stabilize cell membranes along with non-reducing sugars sucrose and trehalose. Cyanobacteria can absorb water many times their dry weight. For example, colonies of *Nostoc* reportedly increase their mean diameter from 50–100 μm to 150–250 μm after wetting. At the same time, cyanobacterial filaments are extruded out of the sheaths, to be retracted inside when the general moisture level decreases [124].

One of the strongest pieces of evidence for the role of EPSs in water stress tolerance was provided by *N. commune* by Tamaru et al. [125]. EPS-deprived cells were significantly harmed in their capability to evolve O_2_, and a decrease in cell viability was observed. In addition, EPSs are also thought to confer an increase in freeze tolerance. Cyanobacterial EPSs provide for the structuring of the biofilms, creating preferential flows of water and nutrients. In addition, EPM creates hydrated microenvironments in which the cells are protected from harmful solar radiation and physical harm and represent a source of carbon for heterotrophs. Under laboratory conditions, Knowles and Castenholz [121] proved that EPSs produced by *Nostoc* sp. CCMEE 6160 improved water stress tolerance of the naturally co-habiting microalga, *Chlorella* sp. CCMEE 6038, which does not produce EPSs.

In biological soil crusts (BSCs), Because EPSs are involved in water capture from both rainfall and non-rainfall sources, crust-covered soils have higher water content than their naked nearby equivalents. The abundance of EPSs was proven to be positively correlated with the water capture capability of the biological crusts. In addition, a significant difference in water-retaining capability after treating soil crust samples for EPM removal was detected. Following a significant water introduction, the swelling of the EPSs is reported to cause soil pore clogging, possibly leading to water run-off [126].

The EPSs intervene in preserving the stability of the membrane vesicles during cycles of drying and swelling, as well as stabilizing desiccation-related enzymes and molecules [99]. As an example, the addition in vitro of the EPSs of *Nostoc commune* CHEN to membrane vesicles prevented them from fusing, counteracting one of the unwanted outcomes of the rehydration process [17].

### Bio-weathering processes

The excretion of EPSs is also key in lithic substrate colonization by epilithic and endolithic cyanobacteria and in the following bio-weathering processes. Surface-dwelling populations endure more UV, temperature, and water stress, which can be mitigated by colonizing subsurface niches. The capacity to modify stone surfaces is owing to their ability to adhere and penetrate within the rock pore spaces, causing exfoliation of the upper substrate layers and irreversible unaesthetic discoloration owing to pigment release. Several investigations aimed at defining the role of EPSs in the fouling caused by cyanobacterial colonization of stone artwork, to elaborate potential control strategies [127].

About 20–30% of stone deterioration has reportedly a biological origin. Stone weathering is carried out by microorganisms by penetrating and pushing apart the cracks in the mineral substrate through cycles of drying/swelling and warming/cooling. By swelling when wetted, the mucous secretions exert great pressure from within. At the same time, mineral dissolution takes place following the release of acidic compounds, Ca^2+^, OH^−^, and organic ligands [128].

In the first rock layers, EPM can concentrate metal cations and nutrients present at low concentrations, sequestering them directly from the substrate. Welch and Vandevivere [129] showed how microbial EPSs enhance the dissolution of feldspathic substrates while forming complexes with framework ions in solution. Indeed, in biofilms, electrostatic interactions produced by cations provide cohesion. Cations serve both as cross-linkers in the biofilm matrix and stimulate physiology-dependent attachment processes in microbial cells by acting as cellular cations and enzyme cofactors [17].

In a recent study, *Plectonema*, *Gloeocapsa*, *Gloeocapsosis,* and *Leptolyngbya* strains isolated from epilithic biofilms showed a good affinity for Ca, Mg, and Fe cations, although to different extents [120]. Divalent cations Ca and Mg cations form cross-bridges with the charged fractions of the EPS strands, increasing the cohesion of the secretions [127]. Additionally, the capability of selectively-immobilize toxic heavy metals could represent a defensive strategy to prevent them from reaching the cells [73, 130].

Generally, marine EPS can play a variety of functions such as adhesive, structural, protection against abiotic stress, bio weathering processes, gliding motility, and nutrient repositories in phototrophic biofilms or biological soil crusts

### Gliding motility of cyanobacteria

Gliding motility requires contact with a solid surface and occurs in a direction parallel to the long axis of the cell or filament. Although the mechanistic basis for gliding motility in cyanobacteria has not been established, recent ultrastructural work has helped to identify characteristic structural features that may play a role in this type of locomotion. Among these features are the distinct cell surfaces formed by specifically arranged protein fibrils and organelle-like structures, which may be involved in the secretion of mucilage during locomotion [17].

Cyanobacteria secrete slime while gliding. That was observed that the EPSs are extruded through junction pore complexes (NPCs), which are prokaryotic organelles with diameters ranging from 70 to 80 nm and 32 nm long, spanning the cell wall. A linked channel, 13 nm in diameter, spans the peptidoglycan layer. In *Phormidium uncinatum* and *Anabaena variabilis*, JPCs are located near the cell septa, at angles of 30-40°, related to the cell axis. Slime extrusion likely propels the cell forward [131]. Oscillin, a Ca-containing protein on the surface of *Phormidium* sp., possibly determines the channels that direct the EPS flow. If oscillin is arranged elliptically, the cell will rotate; if the filaments are arranged radially, the cell will not rotate [132]. Thirty to 40° related to the cell axis. Slime extrusion likely propels the cell forward [131]. Oscillin, a Ca-containing protein on the surface of *Phormidium* sp., possibly determines the channels that direct the EPS flow. If oscillin is arranged elliptically, the cell will rotate; if the filaments are arranged radially, the cell will not rotate [132].

### As effective carbon sources

The composition of the producing community fraction, environmental conditions, and biochemical processes at the community level influence the chemical and physical characteristics of the EPSs. In oligotrophic conditions, EPSs represents a notable source of organic C available for cross-feeding processes. By these means, the activity of the producing organisms is balanced by the activity of the consumers, whereas C from EPSs is the primary substrate respired after rainfall events in deserts. In the aquatic environment, the nutrients can interact with EPSs in order to increase the rate of substance uptake concentrating the dissolved organic compounds to become available to support the microbial growth. In the aquatic environment, the nutrients can interact with EPSs to increase the rate of substance uptake concentrating the dissolved organic compounds to become available to support microbial growth. In the aquatic environment, the nutrients can interact with EPSs to increase the rate of substance uptake concentrating the dissolved organic compounds to become available to support microbial growth. The secretion of EPSs affects many ocean processes [106].

### As cryoprotectant in Arctic areas

Bacteria are found in abundance in the bottom layers of the ice or brine channels and are often attached to detrital particles or to living microalgal cells. In addition, the high numbers of particle-associated bacteria found in sea ice may explain observations of underlying seawater being enriched in bacterial biomass relative to the open ocean. In particular, the EPS may have a cryoprotective role in brine channels of sea ice, where extremes of high salinity and low temperature impose pressures on microbial growth and survival [17].

Particularly, some investigations of sea ice microbial communities have found bacteria strongly associated with particles and have pointed out, as mentioned before, that microbial EPSs played an important role in cryoprotection [92].

EPSs from extremophiles, such as sea ice-microbial communities, ensure their function in strong particle attachment and, more significantly, cryoprotection [92]. This EPSs defend cells from harsh external environmental conditions such as high and low temperature, salinity, radiation, high and low pH, and so on. Extremophiles can thus endure the harmful effects of severe conditions thanks to their EPS coating [10]. EPS produced at 2°C and 10°C had a higher uronic acid content than that produced at 20°C. The availability of iron as a trace metal is of critical importance in the Southern Ocean, where it is known to limit primary production. Exopolymer in the brine channels might have provided buffering against harsh winter conditions and high salinity as well as cryoprotecting the microbes living there against ice crystal formation by depressing the ice nucleation temperature of water [109]. The availability of iron as a trace metal is of critical importance in the Southern Ocean, where it is known to limit primary production. Exopolymer in the brine channels might have provided buffering against harsh winter conditions and high salinity as well as cryoprotecting the microbes living there against ice crystal formation by depressing the ice nucleation temperature of water [109].

Large amounts of microbially generated EPSs have been found in sea ice and along the ice-water contact in Arctic locations [109]. Although diatoms were considered to dominate EPS generation in this system, this material was favorably associated with bacterial abundances. With its high polyhydroxy content, high concentrations of EPSs would lower the freezing point of water in low-temperature, high-salinity brine channels, especially near the cell, where exopolymer concentrations are highest [109]. Arctic sea ice in winter showed that even at temperatures as low as 20°C and salinity of 209 parts per thousand, active bacteria were found in the brine channels and were particle-associated [92]. As well, Mancuso-Nichols et al. [133] studied EPSs produced by sea-ice isolates that were shown, by molecular weight analysis, to be between 5 and 50 times larger than the average observed for other marine EPSs. 2004). As well as, Mancuso-Nichols et al. [133] studied EPSs produced by sea-ice isolates were shown, by molecular weight analysis, to be between 5 and 50 times larger than the average observed for other marine EPSs.

Mancuso-Nichols et al. [47] isolated a strain of Antarctic *Pseudoalteromonas* from sea ice that produced 30 times as much EPS at 2 and 10 °C compared with 20°C in liquid culture. Generally, members of this genus are among the dominant bacteria found in this environment as determined by cultivation-dependent and independent techniques [134]. The finding of Mancuso-Nichols et al. [47] supports the proposed hypothesis that EPS production by psychrotolerant bacteria may play an important role in the sea-ice microbial community. Whether this increased EPS production at low temperature is a specific cold adaptation mechanism for this strain requires further investigation. In addition, the EPS from *Pseudoalteromonas* strain CAM025 is polyanionic and may bind dissolved cations such as trace metals, and therefore the presence of bacterial EPS in the Antarctic marine environment may have important ecological implications [133]. Furthermore, EPS produced by some Antarctic bacterial isolates contain uronic acids and sulfate groups and may act as ligands for cations present as trace metals in the Southern Ocean environment, enhancing the primary production of microbial communities usually limited by poor availability of trace metals such as iron (Fe^+3^) [15, 135]).

Further studies focusing on the biotechnological potential of EPSs produced by bacteria from the Antarctic marine environment have been reported in the literature to date. *Pseudoalteromonas antarctica* NF3 produces an exopolymeric compound of glycoprotein character that displays the ability to coat liposomes and provides protection against surfactants. Even among closely related strains, EPSs produced by Antarctic bacteria commonly found in the marine environment were diverse [133].

### Role in deep sea

Deep-sea hydrothermal vents result from oceanic plate tectonic and submarine volcanic activities. At depths of 500 to 4000 m, they can be found at sea ridges or on subduction back-arc zones. Seawater is charged with metals and other substances such as hydrogen sulfide, hydrogen, ammonia, and carbon dioxide pouring out of chimneys made of precipitates at high temperatures (up to 350°C). The plume appears in varied intensities of white or black hue (white or black smokers) according to the fluid composition [136]. These habitats are transient due to crustal volcanic activity [137]. Some other active areas with a diffuse emission of warm or cold water also exist. Deep-sea ecosystems also include cold seeps and sediments or microbial mats [138].

Until subsurface hydrothermal systems were identified along mid-ocean ridges at depths greater than 2200 m, the deep sea (>1000 m) was assumed to be a biological desert [139]. Hot fumaroles, springs and sediments, and deep-sea vents are examples of geologic formations. Temperatures can range from 380°C within the fumarole to 2°C in the surrounding seawater in these conditions, where hydrostatic pressure averages 25×106 P and temperatures can range from 380°C within the fumarole to 2°C in the surrounding seawater [140]. The vents allow hot anaerobic waters rich in hydrogen sulfide and heavy metals to escape and mix with cold oxygenated seawater. The presence of heavy metals is a characteristic of the hydrothermal vent environment. Despite these environmental extremes, a complex food web based on chemosynthesis, including dense invertebrate populations supported by a rich microbial community of heterotrophic and autotrophic bacteria, was found near the vents [17].

Over the last few decades, these vent communities have yielded an increasing number of new genera and species of both deep-sea hyperthermophilic and mesophilic bacteria. Bacteria associated with deep-sea hydrothermal vent communities have demonstrated their ability to produce unusual extracellular polymers in an aerobic carbohydrate-based medium, and so far, 3 main EPS-producing genera have been identified: *Pseudoalteromonas*, *Alteromonas*, and *Vibrio* [114]. Surprisingly, strains isolated from deep-sea hydrothermal vents showed resistance to heavy metals. Their purified EPSs presented the capacity to bind metals and toxic substances [135].

## Applications of microbial EPSs related to the marine environment

As previously mentioned, many EPSs have revealed interesting chemical compositions and so they are widely used in biotechnological applications and several industries in the different fields of medicine, foods, cosmetics, etc. [141]. As shown in Table 2, there are several examples of commercial microbial EPSs that entered the market such as dextran (produced by labs such as *Leuconostoc mesenteroides*, xanthan gum (the EPS from *Xanthomonas campestris*, and curdlan (produced by *Alcaligenes faecalis*) [27]. Only, this context will offer applications related to the marine environment and aquatic resources. Natural products have long been regarded as important sources of possible chemotherapeutic medicines. Examinations were expanded to include maritime territories in the search for new bioactive chemicals, *mesenteroides*, xanthan gum (the EPS from *Xanthomonas campestris*, gellan (produced by *Pseudomonas elodea*), and curdlan (produced by *Alcaligenes faecalis*) [27]. Only, this context will offer the applications related to marine environment and aquatic resources. Natural products have long been regarded as important sources of possible chemotherapeutic medicines. Examinations were expanded to include maritime territories in the search for new bioactive chemicals.

**Table 2:** A side of the most commercial microbial exopolysaccharides (modified from Mishra and Jha [142])

EPS name	Example of producing microbe
Acetan	*Acetobacter xylinum*
Alginate	*Azotobacter vinelandii*
Cellulose	*Acetobacter xylinum*
Chitosan	*Mucorales* spp.
Curdlan	*Alcaligenes faecalis* var. *myxogenes*
Dextran	*Leuconostoc mesenteroides*
Emulsan	*Acinetobacter calcoaceticus*
Gellan	*Aureomonas elodea*
Glucuronan	*Sinorhizobium meliloti*
Hyaluronic acid	*Streptococcus equi*
Kefiran	*Lactobacillus hilgardii*
Levan	*Alcaligenes viscosus*
N-Acetylglucosamine	*Streptococcus epidermidis*
Pullulan	*Aureobasidium pullulans*
Schizophyllan	*Schizophylum commune*
Scleroglucan	*Sclerotium rolfsii*
Stewartan	*Pantoea stewartii subsp. Stewartii*
Succinoglycan	*Alcaligenes faecalis* var *myxogenes*
Xanthan	*Xanthomonas campestris*

### As anti-fouling agents

Biofouling is a special class of organic fouling and is the result of complex interactions between the substrate, dissolved substances, and microorganisms [143]. Usually, it is ascribed to the accumulation of microorganisms such as bacteria, algae, and fungi on hard surfaces forming the harmful biofilms, via a multi-step and complex formation process [100]. Indeed, the biofouling is one of the most serious problems in marine as a whole and specifically in seawater desalination because it is a very costly problem, keeping busy a billion-dollar industry providing biocides, cleaners, and anti-fouling materials worldwide [100, 144].

Marine biopolymers including EPSs and chitosan EPS may be an effective inhibitor of the initial stages of biofilm formation and subsequent biofouling activity [145]. Under their wide range of metabolic activities and unique structural moieties, marine microbes are an important potential source of anti-fouling compounds. Chemical compounds can affect the bacterial structure of the biofilm and interfere with the larval settlement of higher organisms [143].

The EPS producers isolated from hydrothermal vents are relatively non-fastidious (aerobes, mesophiles, heterotrophs) [114]. Under laboratory conditions, some bacteria from these environments produce large amounts of EPSs, which offer massive potential for the exploitation of antifoulants. They showed strong anti-microbial and anti-fouling activities [143]. However, the EPSs do not contain toxic heavy metals or other molecules that adversely affect the local ecology. In addition, they can easily be produced using relatively simple bacterial cultivation protocols and commercially available fermentation equipment [106].

These polymers may be able to inhibit the larval settlement of marine macrofoulers in a non-toxic version to some extent. As a result, EPSs used as a permanent coating on other organic films may affect biofilm formation by preventing bacterial adhesion in naturally flowing seawater. Recent research has found that some EPSs when used at very low concentrations, can prevent bacterial adhesion and the formation of an active biofilm. The possibility of EPS inhibiting microfouling via steric hindrance mechanisms should be investigated further [146].

### In wastewater treatment

Microbial EPSs can adsorb metal cations, as well as other dissolved substances, which can aid in heavy metal bioremediation. This could be useful in wastewater treatment systems. Biofilms can bind to and remove metals like copper, lead, nickel, and cadmium, for example. The metal specificity and binding affinity of EPS vary depending on polymer composition and environmental factors [147].

On the other hand, the flocculation step is considered a vital stage during the treatment of raw water from pollutants. It helps in the removal of dissolved organic substances and turbidity from water through the addition of chemical coagulants such as alum, ferric chloride, and synthetic organic polymers [148]. These coagulants have some drawbacks, including ineffectiveness in cold water, high procurement costs, complete or partial non-biodegradability, human health effects, the production of large amounts of sludge, and a significant impact on the pH of treated water. Furthermore, a direct link between the use of these chemical coagulants and the development of Alzheimer’s disease has been established [149]. In addition, partial degradation of synthetic coagulant polymers produces intermediate substances, which have some neurotoxic and carcinogenic effects [150]. Therefore, searching for alternative natural-based coagulants to avoid these disadvantages becomes an insistent issue. Natural coagulants were applied in water treatment and showed many advantages such as low cost, low toxicity, biodegradability, and small volumes of sludge [150].ts were applied in water treatment and showed many advantages such as low cost, low toxicity, biodegradability and small volumes of sludge [150].

Many natural coagulants are produced from microorganisms are composed of bio-macromolecules such as polysaccharides, proteins, lipids, and nucleic acids [151]. Most studies focused on the removal of only one type of pollutants using microbial coagulants such as heavy metals or dyes [152], while no more reports about multiple pollutants removal [153]. Many EPS-producing bacteria have been discovered in extreme marine environments with high levels of toxic elements such as sulfur and heavy metals. As a result, the EPSs they produce have a strong affinity for heavy metals and could be widely used in the bio-detoxification and wastewater industries to remove heavy metals. Additional rheological studies showed the uronic-rich EPS could be expected to have the ability for some heavy metal-binding and therefore applied in the bio-detoxification and wastewater treatment [17] or dyes [152], while no more reports about multiple pollutants removal [153]. Many EPS-producing bacteria have been discovered in extreme marine environments with high levels of toxic elements such as sulfur and heavy metals. As a result, the EPSs they produce have a strong affinity for heavy metals and could be widely used in the bio-detoxification and wastewater industries to remove heavy metals. Additional rheological studies showed the uronic-rich EPS could be expected to have ability for some heavy metal-binding and therefore applied in the bio-detoxification and wastewater treatment [17].

For example, the EPS formed by *Alteromonas macleodii* sub. sp, *fijiensis* also has this property. The viscosity of this EPS had the same order of magnitude of a commercial xanthan [154]. The native EPS, produced by *A. infernus,* shows a very strong affinity for heavy metals such as; Pb, Cd, and Zn. In addition, the EPS secreted by *Cyanothece* sp. ATCC 51142 is highly effective for metal removal from solutions and can remove different metals from industrial wastes [155]. The EPS produced by *Alteromonas* sp. strain 1644 showed strong selectivity between monovalent and divalent ions and exhibited a great affinity for divalent ions, such as Mg cations [156].

On the other side, synthetic flocculants used in wastewater treatment plants, such as Al_2_SO_4_ and poly-AlCl_2_ and organic synthetic polymers of polyacrylamide derivatives and polyethylene imine, have been known to possess adverse health effects such as; carcinogenicity, neurotoxicity and Alzheimer’s disease [157]. So, the microbial EPSs as flocculants with various properties were effectively applied as safe alternatives for chemical flocculants. Bioflocculants have been expected to be harmless to the environment because of their biodegradability [158]. Several workers have reported high flocculation efficiency mediated by the EPSs produced by *Sorangium cellulosum* NUST06, *Virgibacillus* sp., *Bacillus* sp., and *Artrobacter* sp., which were isolated from fresh and marine waters [159, 160].

In addition, heavy metals adsorption by microbial EPSs is widely reported by other strains such as *Bacillus firmus* [161] and *Paenibacillus validus* MP5 [152]. Al-Wasify et al. [162] used EPSs from *Bacillus licheniformis*, *B. insolitus,* and *B. alvei* as natural coagulants during the coagulation-flocculation process. They discovered that when extracted EPSs were used as sole coagulant materials, they had a high removal efficiency and that when alum was added to bacterial EPSs, the removal efficiency increased. Recently, Szewczuk-Karpisz & Wiśniewska [163] studied the *Sinorhizobium meliloti* 1021 EPS flocculation efficiency relative to mineral oxide suspensions (Cr_2_O_3_, SiO_2_, and ZrO_2_). Their data verified the application of *S. meliloti* EPS in wastewater treatment as a potential flocculant related to these solids.

The high removal efficiencies of the studied microbial EPSs as natural coagulants, on the other hand, may be attributed to strong adsorption with positive charges carrying metals such as heavy metals, debris, oily particles, organics, and mud, resulting in the formation of large-sized and heavy-weight flocs. During rapid and slow water mixing, these new flocs grew in size. This phenomenon allows rapid degradation of organics in water which decreases levels of organic pollution in water, turbidity level, and other related physicochemical parameters [17].

### In bioremediation field

In the beginning, bioremediation is considered one of the most common applications for EPSs in many fields related to the marine environment [164]. This occurs because EPSs contain many functional groups, such as amine, phosphate, hydroxyl, carboxyl, and urinate, which increase the negative charge of EPSs and their ion exchange properties and flocculation activities, as well as the ability to coordinate with metal ions and form organic precipitation [165]. Furthermore, due to the labile nature of microbial EPSs and their ability to bind heavy metals, the bound metals are routed through the marine food chain, assisting in the bioaccumulation of metal pollutants in higher trophic animals [166]. Furthermore, due to the labile nature of microbial EPSs and their ability to bind heavy metals, the bound metals are routed through the marine food chain, assisting in the bioaccumulation of metal pollutants in higher trophic animals [166].

Therefore, one of the most essential applications of EPSs is the bioremediation of targeted pollutants such as heavy metals, polycyclic aromatic hydrocarbons, petroleum, nitroaromatics, polycyclic aromatic hydrocarbons, polychlorinated biphenyls, chlorinated phenols, and aliphatics [167].

One of the mechanisms by which organisms remove or accumulate heavy metals is biosorption. It is a fast and passive metal uptake process where the cells do not need to be alive. Adsorption, absorption, intracellular or extracellular accumulation, redox reaction, ion exchange, surface complexation, and precipitation are some of the mechanisms involved in biosorption [168]. Microbial EPS can bind with anion and cations, resulting in a candidate of choice for the bioremediation process [169]. In some remediation processes, EPS modified by chemical processes such as acetylation, methylation, phosphorylation, and sulfonylation are used [170]. Acetylation of EPS decides the selectivity of metal-binding [171]. The metal binding property of the EPS plays a significant role in metal remediation from wastewater [172].

The reports of Gupta & Diwan [173] demonstrated almost 85–95% of zinc, copper, and chromium removal using a consortium developed from activated sludge. They also reported that many Gram-negative bacterial consortia could remove 75–78% of zinc, lead, chromium, nickel, copper, cadmium, and cobalt within two hours. Immobilized EPS of *Chryseomonas* and *Paenibacillus* polymyxa showed the removal of cadmium, cobalt, copper, and lead [174, 175]. Dead cell-bound EPS of *Bacillus cereus*, *Bacillus pumilus*, and *Pentoea agglomerans* showed 85.5–89% of chromium removal [176]. EPS of *Acidithiobacillus ferrooxidans* helps the organisms to bind with the mineral and thus extract metals from the sulfide ores [177]. Salehizadeh and Shojaosadati [178] reported the biosorption of copper (74.9%), lead (98.3%), and zinc (61.8%) by the EPS of *Bacillus firmus*. The EPS produced by *Azotobacter chroococcum* XU1 showed the sorption of lead (40.48%) and mercury (47.87%) [179]. The EPS of *Ensifer meliloti*, showed 89, 85, and 66% of lead, nickel, and zinc ion reduction, respectively [180]. Various marine bacteria are also reported for their metal removal ability. The specific structure and high uronic acid content impart an enhanced anionic property to marine bacterial EPS, which may be responsible for metal removal. EPS of Marinobacter sp. showed sorption of metals like lead and copper [166]. EPS from marine *Enterobacter cloacae* demonstrated the sorption of cadmium (65%), copper (20%), and hexavalent chromium (75%) [181]. *Halomonas* sp. associated with marine microalga was also reported to chelate metals such as calcium, aluminum, iron, and magnesium [45]. The EPS secreted by the *Pseudoalteromonas* sp. SM9913 showed the adsorption of Fe^2+^ (85.00%), Zn^2+^ (58.15%), Cu^2+^ (52.77%), Co^2+^(48.88%), Mg^2+^ (30.69%), Mn^2+^ (25.67%), and Cr^6+^ (5.15%) [182].

Because biofilm-mediated bioremediation is an effective and safe method for removing pollutants from water [183], apart from this, it is also used to enhance oil recovery [115]. As well as, some special applications like sludge settling and dewatering were demonstrated with EPSs [184]. However, their amazing examples that support the application of microbial EPSs in the bioremediation field, are as follows:

i. EPSs of *Hansenula anomala* CCY 38-1-22 bound 90% of the total amount of Cd ions absorbed by this resistant strain, while the sensitive strain of *Saccharomyces cerevisiae* CCY 21-4-100 accumulated this metal predominantly in the cellular compartments [185].
ii. Fungal EPS from *Flavodon flavus* may serve in the degradation of toxic organic compounds by breaking down polycyclic aromatic hydrocarbons [186], Kumar et al., [97].
iii. Each gram of pestan, a specific EPS produced by *Pestalotiopsis* sp. KCTC 8637, can absorb 120 mg of lead or 60 mg of Zn [187].
iv. Pullulan extracted from *Aureobasidium pullulans* CH1 strain, was reported to bioadsorb metal (Cu, Fe, Zn, Mn, Pb, Cd, Ni, and Cr) [188].
v. Sulfated EPS secreted by a bacterium isolated from marine microbial mats has a very high affinity for binding to Cu and Fe [189].
vi. EPS produced by the fungus; *Colletotrichum* sp. contributed to the removal of Cd and Pb ions by biosorption [167].

### In petroleum industry

The petroleum industry, amazingly, uses bacterial xanthan gum in oil drilling, fracturing, and pipeline cleaning, and due to its excellent compatibility with salt and resistance to thermal degradation, it is advantageous as an additive in drilling fluids [102]. Xanthan gum outperforms other polymers in terms of viscosity, thickening, salt resistance, and contamination resistance; especially in the good drilling of sea, beach, high halide layer, and permafrost layer, xanthan gum has a remarkable effect in sludge treatment, completion fluid, and tertiary oil recovery, as well as a significant function for accelerating drilling speed and preventing thawing. This product, as a kind of ideal additive, has a bright future ahead of it [17].

The rheological characteristics of xanthan gum were measured in linear core flow tests. This constitutive flow behavior was used in a radial flow simulator to predict the invasion profile of xanthan gum in the formation. Radial flow tests were performed to validate the predictions from the simulator and to observe the effect of fluid loss additives such as starch and ground Berea. Therefore, xanthan gum has already been used in the different stages of the oil industry such as; the drilling industry; because its functions are adding viscosity and shearing force, improving the suspending power of drilling fluid which is essential in using functions of the drilling fluid, oil exploitation industry; due to it contains many essential conditions required for improving oil recovery rate. Xanthan gum is an excellent additive for oilfield drilling mud, and in the oil industry compared with polyacrylamide, carboxymethylcellulose, modified starch, and some plant polysaccharides, etc. has a clear technological advantage in oilfield development for its high ability to increase the viscosity, thickening, anti-salt, and anti-pollution [17].

Further, the rheological properties of the EPS secreted by the halophilic archaebacterium; *Haloferax mediterranei* showed a pseudoplastic behavior and a high apparent viscosity at relatively low concentrations and this viscosity is remarkably resistant to extremes of pH, temperature, or salinity. These characteristics make this EPS to be used for enhanced oil recovery and other applications, which require a very resistant thickening agent [190].

On the other side, microbial EPSs are used as biosurfactants and bioemulsifiers that attracted great attention because of their biodegradability [115]. Therefore, there is a promising technology for manipulating the function and structure of microbial environments existing in oil reservoirs known as; microbial enhanced oil recovery (MEOR). It is a biotechnology branch in which microbes are found to recover the additional oil from existing wells, thereby enhancing the petroleum production of an oil reservoir [115]. However, selected natural microbes producing bio-surfactants and/or specific EPSs are introduced into oil wells to produce harmless by-products, such as slippery natural substances or gases, all of which aid propel oil out of the well allowing a more amount to be recovered from the well. Genetically engineered *Enterobacter cloacae* are successfully used in MEOR [115].

## Conclusion

The information provided in this review supports some general conclusion points regarding the characteristics of the EPSs produced by marine microbes and their roles, functions, and applications in the marine environment:

1. Increasing awareness of the environment and green technology might enable the use of microbes as a renewable and alternative resource of EPSs instead of synthetic and other EPSs.
2. Many marine microbes are promising resources for producing EPSs that can provide significant opportunities for newer roles, functions, and applications.
3. Cyanobacteria and microalgae can produce more complex EPSs than other EPS-producing microorganisms.
4. Different types of marine and extremophilic microbes should be further explored to harness their superior characteristics for creating novel EPSs with higher productivity and unique applications.

## Data Availability

All data of the current study are included in this article.
